# Copeptin and Mid-Regional Proadrenomedullin Are Not Useful Biomarkers of Cardiometabolic Disease in Patients with Acromegaly—A Preliminary Study

**DOI:** 10.3390/biomedicines13030666

**Published:** 2025-03-08

**Authors:** Martyna Strzelec, Eliza Kubicka, Justyna Kuliczkowska-Płaksej, Katarzyna Kolačkov, Łucja Janek, Marek Bolanowski, Aleksandra Jawiarczyk-Przybyłowska

**Affiliations:** 1 Department and Clinic of Endocrinology and Internal Medicine, Wroclaw Medical University, Wybrzeże Pasteura 4, 50-367 Wroclaw, Poland; stulamartyna@gmail.com (M.S.);; 2 Department of Nuclear Medicine, Tadeusz Marciniak Lower Silesia Specialist Hospital-Centre for Medical Emergency, 54-049 Wroclaw, Poland; 3 Statistical Analysis Centre, Wroclaw Medical University, 50-368 Wroclaw, Poland

**Keywords:** acromegaly, cardiovascular diseases, copeptin, mid-regional proadrenomedullin

## Abstract

**Background/Objectives:** Cardiovascular complications are a leading cause of premature mortality in patients with acromegaly. Copeptin (CPP) correlates strongly with plasma osmolality and is regulated by non-osmotic stimuli involved in the pathophysiology of cardiovascular disease. Mid-regional proadrenomedullin (MR-proADM), synthesized mainly in the adrenal medulla, vascular endothelial cells, and the heart, has vasodilatory effects. The study aimed to assess two cardiovascular biomarkers (CPP and MR-proADM) in acromegaly patients in relation to disease activity and compare findings with a control group. **Methods:** The study examined CPP and MR-proADM levels alongside hormonal and biochemical parameters and cardiovascular and metabolic disease prevalence in 53 acromegaly patients and 26 controls. **Results:** No significant differences in CPP or MR-proADM concentrations were observed between the two groups. However, a positive correlation occurred between growth hormone (GH) and CPP concentrations, and there was a negative correlation between fasting glucose and CPP concentrations in acromegaly patients. The study also found a positive correlation between low-density lipoprotein (LDL) cholesterol and MR-proADM concentrations and between high-density lipoprotein (HDL) cholesterol and MR-proADM levels in the study group. Moreover, atherogenic dyslipidemia was significantly more common in the active form of acromegaly and pituitary macroadenoma patients than in the control group. Acromegaly patients had significantly higher fasting glucose and fasting insulin levels compared to controls, and the homeostasis model assessment of the insulin resistance (HOMA-IR) index was significantly lower in the study group than in the controls. **Conclusions:** Neither CPP or MR-proADM are significant diagnostic or monitoring indicators of cardiovascular or metabolic complications in acromegaly.

## 1. Introduction

Acromegaly is a rare chronic endocrinopathy caused by excessive growth hormone (GH) secretion, most commonly by a pituitary adenoma (PitNET, pituitary neuroendocrine tumors). Excessive GH levels and, secondarily, insulin-like growth factor 1 (IGF-I) cause a range of complications, from changes in appearance to soft tissue and bone hypertrophy, leading to systemic disorders that result in shortened survival rates and reduced quality of life [1,2,3]. Typical consequences of untreated acromegaly include cardiovascular, metabolic, endocrine, respiratory, bone, and oncological complications. Cardiovascular complications are a primary cause of premature mortality in acromegaly patients, responsible for around 60% of deaths [4], though biochemical control of the disease (normalization of GH and IGF-I levels) reduces mortality to that observed in the general population [5,6,7]. The most common cardiovascular diseases (CVD) associated with increased mortality in active acromegaly (AA) include hypertension, cardiomyopathy, heart valve disease (most frequently mitral and aortic), arrhythmias (such as paroxysmal atrial fibrillation, supraventricular tachycardia, sick sinus syndrome, extra ventricular beats, ventricular tachycardia, and left ventricular dyssynchrony), atherosclerosis, and coronary artery disease. The main CVD factors in acromegaly are GH hypersecretion, disease duration, age, nicotinism, obesity, dyslipidemia, insulin resistance, diabetes, and sleep apnea syndrome [8,9,10,11]. The presence of CVD at the time of acromegaly diagnosis increases the hospitalization rate by up to threefold and may account for 60–100% of deaths over a 15-year period [8].

Excess GH leads primarily to insulin resistance and secondarily to hyperinsulinemia, with impaired glucose tolerance prevalence of 16–46% and a diabetes frequency of 20–56% [3]. In AA, decreased fat mass, increased lean body mass, and dyslipidemia (increased concentrations of triglycerides [TG] and low-density lipoprotein [LDL] cholesterol and decreased high-density lipoprotein [HDL] cholesterol levels) are observed [9]. Many studies evaluating cardiovascular and metabolic biomarkers in acromegaly have been reported in the literature, though no clinical utility has been found for assessing levels of adiponectin, total homocysteine, or *H19* ribonucleic acid (RNA) as markers of cardiovascular complications in acromegaly patients [12,13,14]. However, endothelin-1 levels were a vital marker of premature atherosclerosis and a novel risk factor for endothelial dysfunction and early vascular complications [13,14]. Moreover, a relationship was shown between fat mass and obesity-associated (*FTO*) polymorphisms and HDL cholesterol levels, suggesting an association with higher CVD risk in acromegaly patients, and N-terminal pro-B-type natriuretic peptide (NT-pro-BNP) was an independent predictor of cardiovascular events in these patients [15,16]. No previous studies have evaluated copeptin (CPP) or mid-regional proadrenomedullin (MR-proADM) as cardiovascular biomarkers in acromegaly patients.

Any factor that induces stress in the body, including any acute illness, activates the hypothalamic–pituitary–adrenal axis and stimulates the release of hypothalamic stress hormones, such as vasopressin (AVP) [17]. Neurohormonal activation plays a key role in the pathophysiology of CVD. AVP, through its action on the V2 receptor, contributes to the development and progression of chronic kidney disease, which may exacerbate other cardiovascular risk factors [18]. Furthermore, AVP, via the V1a receptor, induces platelet aggregation, has vasoconstrictive effects, and contributes to pathological remodeling of the heart muscle [19]. It also plays a role in glucose homeostasis (through its influence on the stimulation of gluconeogenesis and glycogenolysis in the liver, as well as the release of glucagon or insulin from pancreatic islets), and elevated AVP levels are risk factors for metabolic syndrome and diabetes [20]. Measuring circulating AVP levels is challenging because it is released in a pulsatile manner, is unstable, and is rapidly cleared from plasma [17]. CPP, a 39-amino acid peptide with a molecular mass of approximately 5 kDa, is a stable fragment of the AVP precursor. It is released in equimolar proportion to AVP and appears to be a clinically significant method for reliably assessing the concentrations of this hormone [21]. The literature contains research supporting the association between copeptin levels and cardiovascular risk. The study of two independent French cohorts showed that plasma copeptin was positively associated with major cardiovascular events (myocardial infarction, coronary revascularization, congestive heart failure and cardiovascular death during a 5-year follow-up period) in patients with type 2 diabetes [20]. In addition, a Swedish study confirmed the hypothesis that copeptin predicts coronary artery disease risk and cardiovascular mortality in an older population [22]. Therefore, CPP is considered a potentially valuable diagnostic and prognostic biomarker in cardiometabolic diseases.

Another neurohumoral component regulating the cardiovascular system is adrenomedullin (ADM). This 52-amino acid peptide is synthesized in the adrenal medulla, endothelial cells, and the heart in response to stimulation by various hormones and cytokines. It regulates vascular tone and blood pressure through autocrine and paracrine mechanisms [23]. ADM is a potent vasodilator (acting via the production of cyclic adenosine monophosphate (cAMP) and enhancing nitric oxide (NO) synthesis). It increases myocardial contractility through the activation of protein kinase A (cAMP-dependent mechanism) and by increasing intracellular calcium levels [24]. Furthermore, it protects the tissues of the circulatory system from damage by inhibiting apoptosis, regulating smooth muscle cell proliferation, and exerting antioxidant and anti-inflammatory effects [25]. MR-proADM is a more stable form of ADM. Its prolonged half-life, lasting several hours, ensures resistance to various factors, including age, sex, and diurnal fluctuations, making this hormone a significant cardiometabolic marker with potential for use in new diagnostic and therapeutic purposes [26]. The study of a large European cohort showed that MR-proADM is an independent predictor of mortality in patients with chronic heart failure, and assessment of this biomarker adds prognostic information to N-terminal pro-B-type natriuretic peptide (NT-proBNP) [27].

Both these biomarkers can provide valuable insights into the disease’s complications and clinical management in acromegaly. In acromegaly, patients often have comorbidities such as hypertension, heart failure, and diabetes, so the assessment of copeptin may give additional information regarding the stress burden these conditions impose on the body [3,9,28]. Elevated levels of GH and IGF-I in acromegaly often lead to significant vascular remodeling changes in endothelial function and increased blood pressure [11]. These changes burden the cardiovascular system and other organs, making the monitoring of copeptin a valuable tool for clinicians. Moreover, it could also be used as a prognostic biomarker in emergency settings, including in patients with acromegaly with associated complications (for example, as a result of pituitary surgery, history of ischemic stroke, myocardial infarction, exacerbation of heart failure, or chronic obstructive pulmonary disease) [28,29]. MR-proADM levels are sensitive to vascular remodeling, changes in endothelial function, increased blood pressure and can indicate the degree of vascular damage or stress. Consequently, it could be a useful marker for assessing the severity of hypertension and cardiovascular disease [26,30]. Elevated MR-proADM concentrations could indicate poor prognosis and help assess the cardiometabolic risk in acromegaly patients, especially those with pre-existing heart disease, hypertension, or metabolic syndrome. MR-proADM also plays a role as a marker of inflammation. Moreover, it is elevated in conditions like sepsis and infection [31]. Therefore, it could be a useful biomarker for identifying acromegaly patients at high risk for infectious complications, particularly in the emergency setting and could guide early intervention for patients at risk of sepsis or pneumonia. Together, both biomarkers, with their roles in vascular health, stress response, and metabolic dysfunction, could provide complementary tools for monitoring disease progression, predicting complications, and influencing treatment strategies in acromegaly patients.

The primary study aim was to assess the concentrations of CPP and MR-proADM in acromegaly patients in relation to disease activity and draw comparisons with the control group (CG). In addition, we analyzed carbohydrate and lipid metabolism parameters in both groups of patients and examined their association with CPP and MR-proADM levels.

## 2. Materials and Methods

The study group consisted of 53 acromegaly patients (30 females and 23 males, aged 55.89 ± 14.8) and 26 control subjects (18 females and 8 males, aged 56.23 ± 15.14). The inclusion criterion for the study group was a current or past acromegaly diagnosis, defined as elevated IGF-I levels and unrepressed GH on the oral glucose tolerance test (OGTT), according to current recommendations of the Endocrine Society and the Polish Society of Endocrinology [1,32]. The acromegaly patients were divided into three subgroups: AA, GH ≥ 1.0 µg/L in the OGTT and IGF-I above the age- and sex-specific norm (11 subjects); cured acromegaly (CuA), GH < 1.0 µg/L in OGTT and IGF-I within the age- and sex-specific norm (15 subjects), and controlled acromegaly (CoA), where the constellation of results was similar to that of CuA during pharmacotherapy (27 subjects). In the AA group, four patients had the disease diagnosed de novo before the treatment. Based on the size of the pituitary tumor at the time of acromegaly diagnosis, two groups were classified: macroadenomas (if at least one dimension was ≥10 mm) and microadenomas (<10 mm). The study group was evaluated for the presence of hypopituitarism and acromegaly recurrence.

The CG included 26 patients without somatotropic axis dysfunction and no diseases that could potentially affect CPP and MR-proADM concentrations, with the exception of CVD and carbohydrate or lipid disorders. All participants were diagnosed and treated at the Department of Endocrinology, Diabetes, and Isotope Therapy at Wroclaw Medical University (Poland) from 2018 to 2020. The current and past medical records of all study participants were analyzed for primary medical history and comorbidities. We evaluated the available data for clinical cardiovascular risk factors, the presence of cardiovascular complications (such as hypertension, arrhythmia, heart failure, changes in echocardiograms, coronary artery disease, arterial and capillary disease, history of embolism, and stroke), and metabolic complications (hyperlipidemia, pre-diabetic state, diabetes, insulin resistance, and atherogenic dyslipidemia). The definition of atherogenic dyslipidemia (according to the 2018 Recommendations for the Treatment of Dyslipidemia in Poland) that we used in our study included: triglyceride concentration ≥ 150 mg/dL and decreased HDL cholesterol concentration (respectively, <40 mg/dL in men and <45 mg/dL in women) [33]. We recorded patients’ anthropometric data: body weight (kg), height (cm), and body mass index (BMI) (kg/m^2^), and took blood pressure measurements (mm/Hg).

### 2.1. Laboratory Examinations

Fasting venous blood samples were collected from all study participants, with GH, IGF-I, and insulin concentrations assayed using a chemiluminescence immunometric assay. IGF-I levels were expressed relative to the upper limit of normal (ULN) for the patient’s age. Total cholesterol assays used an enzymatic method with oxidase, esterase, and peroxidase, while LDL cholesterol estimations employed the Friedewald equation, HDL cholesterol measurements used direct precipitation with a combination of polymer polyanions, and TGs were assayed using a routine enzymatic method. Atherogenicity indexes, including Castelli Index 1 and 2, atherogenic index of plasma (API), and atherogenic coefficient (AC), were calculated. For 51 subjects, OGTT was performed, and serum glucose levels were assayed at the 120th minute of the test using a hexokinase method. Insulin resistance indices (homeostasis model assessment of insulin resistance [HOMA-IR] index] and quantitative insulin sensitivity check index [QUICKI]) were also estimated.

Serum CPP and MR-proADM concentrations were determined by enzyme-linked immunosorbent assay (ELISA) using commercially available kits purchased from various suppliers. The serum was separated by centrifugation of blood samples at 1000× *g* for 20 min, then stored in aliquots at −80 °C. Samples were brought to room temperature before analysis. The following kits were used: ELISA Kit for CPP (Catalogue No. CEA365Hu; Cloud-Clone Corp., Katy, TX, USA), human MR-ProADM ELISA Kit (Catalogue No. E3214Hu; Bioassay Technology Laboratory, Shanghai, China). Tests were performed according to the manufacturer’s instructions. The variability of coefficients for CPP are as follows: in the intra-assay CV < 10%, in the inter-assay: CV < 12%. Whereas for MR-ProADM, respectively: in the intra-assay: CV < 8%, in the inter-assay: CV < 10%. The bioethics committee of the Wroclaw University of Medical Sciences approved the study protocol (number 615/2018 on 30 October 2018 and number 120/2024 on 21 March 2024). All participants signed informed consent forms in accordance with the Declaration of Helsinki.

### 2.2. Statistical Analysis

All statistical analyses employed Statistica software (version 13.3.721.1). The Shapiro–Wilk test assessed the normality of distribution for Student’s *t*-test, and if non-normal, Wilcoxon’s test for rank sums for independent samples (Mann–Whitney-U test [M-W test]) examined the median and quartiles. The mean and standard deviation were presented when the normality assumption was met, and the Brown–Forsythe test determined the homogeneity of variance. If there was no homogeneity of variance, Welch’s *t*-test with independent variance estimation was used, while Student’s *t*-test for independent samples was applied when the assumption was satisfied (relative to groups). For nominal variables, the assumption of expected abundance (n < 5 ≤ 20% of cells) was assessed for Pearson’s Chi-square test of consistency (differences). If the assumption of expected abundance was not met, Fisher’s exact test for 2 × 2 tables was used. Normality and linearity assumptions for Pearson’s linear correlation and the tied rank assumption (≥20% rank) for Kendall’s rank correlation were also tested. The assumptions for Pearson’s and Kendall’s correlations were unmet, so Spearman’s rank correlation was used. Generated data determined if the function was monotonic or if there was only a monotonic correlation component. A straight line was fitted based on the ranked values. No correction for multiple comparisons was applied, and the analysis was assumed to be exploratory. An α < 0.05 was considered statistically significant.

## 3. Results

Table 1 presents the general characteristics of the acromegaly patients and controls. Mean body weight and BMI were higher in the acromegaly patients than the CG (the M-W test: *p* = 0.002 and 0.021, respectively). The mean age of acromegaly diagnosis was 41. In the study group, 43 (81.13%) patients were diagnosed with pituitary macroadenoma (if at least one dimension of the tumor was ≥10 mm) at the time of diagnosis, and 10 (18.87%) were diagnosed with microadenoma, with 34 patients undergoing one transsphenoidal neurosurgery and 8 requiring reoperation by the same method. Transcranial access surgery was performed in three cases, with four patients undergoing radiotherapy by conventional methods and four by stereotactic techniques. No surgery or irradiation of the pituitary region was performed in the CG. Thirty-seven (69.81%) acromegaly patients experienced recurrence after unsuccessful surgical treatment. Pituitary insufficiency was diagnosed in 20 (37.75%) patients and 2 (9.52%) controls. There were 12 cases of hypothyroidism of the thyrotropic axis, 11 of the adrenocorticotropic axis, 9 of the pituitary gonadotropic axis, and 1 case of panhypopituitarism. All patients received an adequate dose of hormone replacement.

### 3.1. Copeptin and Mid-Regional Proadrenomedullin

We did not observe significant differences in CPP concentrations between the study group and CG. In the group of acromegaly patients, we found a positive correlation between GH and CPP concentrations (result close to statistical significance *p* = 0.060) with a weak trend (ρ = 0.27) (Table 2). Analyzing the parameters of carbohydrate and lipid metabolism found a statistically significant (*p* = 0.025) negative correlation with a very weak trend (tau c = −0.11) between fasting glucose and CPP concentrations in the study group (Table 3).

There were no statistically significant differences in MR-proADM concentrations between the acromegaly patients and the CG. No significant correlations were obtained between MR-proADM levels and GH or IGF-I × ULN concentrations in the study group, though we observed a positive correlation with a weak trend (tau c = 0.24) between LDL-C and MR-proADM concentrations (*p* = 0.010) in the acromegaly patients (Table 4). In addition, the study group had a positive correlation between HDL-C concentration and MR-proADM concentration (*p* = 0.017, tau c = 0.23). No other significant correlations were recorded for cardiovascular biomarker levels (CPP and MR-proADM) or carbohydrate (fasting glucose, glucose at 120 min OGTT, insulin, HOMA-IR index, and QUICKI) and lipid metabolism (total cholesterol, HDL cholesterol, LDL-C, TGs, Castelli Index 1 and 2, API, and AC) in the CG.

### 3.2. Comorbidities

The prevalence of comorbidities is presented in Table 5.

Hypertension, changes in echocardiograms, and arterial and capillary disease were the most common cardiovascular complications in patients with acromegaly, with hypertension diagnosed in more than half of the study group. The most frequent changes in echocardiograms in acromegaly patients included mild valvular regurgitation (nine cases, including seven mitral, six aortic, five tricuspid, and three pulmonary), myocardial hypertrophy (eight cases), and atrial cavity enlargement (six cases, including six left and one right). This was followed by valvular stenosis (two cases, including one aortic and one mitral), diastolic dysfunction (two cases), and dilation of the ascending aorta (one case). Of the metabolic complications in the study group, hyperlipidemia dominated, with a frequency of almost 70%; others occurred with comparable frequency in one in three acromegaly patients. CVD in the CG was dominated by hypertension, with hyperlipidemia the most common metabolic disorder in this group. We did not observe differences in the prevalence of cardiovascular or metabolic complications in either group.

Analyzing the effects of acromegaly activity on the occurrence of cardiovascular and metabolic disorders demonstrated that atherogenic dyslipidemia was significantly more common in AA than in the CG (*p* = 0.046) (Table 6), though it had no effect on other complications. The presence of hypopituitarism had a non-significant effect on the prevalence of cardiovascular, lipid, and carbohydrate metabolism disorders in the study group, and atherogenic dyslipidemia was significantly more common in patients with acromegaly due to pituitary macroadenoma than in the controls (*p* = 0.046). However, pituitary tumor size did not significantly affect the other analyzed comorbidities in the study group.

Acromegaly patients displayed significantly higher fasting glucose (*p* = 0.037) and fasting insulin concentrations (*p* = 0.043) than the CG, though the opposite was found in the HOMA-IR index analysis (*p* = 0.054). Neither group varied in glucose concentration for the 120 min OGTT or QUICKI, and there were no differences in total cholesterol, LDL cholesterol, HDL cholesterol, TG, Castelli index 1 and 2, API, or AC in either group.

## 4. Discussion

The hormones of the GH/IGF-I axis have a significant impact on the cardiovascular system. GH increases the mass and contractility of the heart muscle, reduces vascular resistance, and improves endothelial function. Its anabolic properties include enhanced synthesis of cardiac muscle proteins and reduced apoptosis [9]. Both acromegaly and GH deficiency are associated with increased morbidity and mortality from cardiovascular causes. Adults with GH deficiency, in the absence of replacement therapy, exhibit reduced left ventricular mass, lower cardiac output, decreased preload, and lower exercise capacity. Additionally, they present with accelerated atherosclerosis, insulin resistance, unfavorable changes in lipid profiles, including elevated serum LDL cholesterol levels, impaired fibrinolysis, and increased sympathetic nervous system activity. Growth hormone replacement therapy normalizes most of the cardiovascular risk factors observed in patients with hypopituitarism [34].

It is important to remember that the GH-IGF-I axis can be affected by various factors, such as starvation, feeding, eating habits, stress, diabetes, or kidney failure [35]. Hormones of the pituitary somatotropic axis have a protective effect on the heart, and reduced levels of IGF-I have been observed in patients with systolic dysfunction, in patients after myocardial infarction, and in elderly patients at risk of heart failure. The resistance to GH with reduced IGF-I levels may be related to the process of catabolism (muscle weakness, loss of muscle mass, development of cachexia) in chronic heart failure (CHF). Petretta et al. in their study indicated that NT-proBNP and IGH-I/GH ratio may be useful as independent predictors of death in patients with CHF [36]. Similarly, in a study by Fröhlich et al., patients with advanced cancer showed reduced left ventricular mass, associated increased plasma GH levels, and a reduced IGF-I/GH ratio, indicating increasing acquired resistance to GH, especially in patients with cachexia syndrome [37]. The literature contains studies analyzing the use of GH therapy as a promising treatment option for heart failure due to the effect of improving left ventricular ejection fraction, exercise capacity, and reducing inflammatory markers [38].

Acromegaly is linked to an increased risk of cardiac and metabolic complications, as has been shown in numerous studies [39,40]. The presence of any CVD at the time of acromegaly diagnosis can triple the probability of hospitalization and account for as much as 60–100% of deaths in this group of patients over a 15-year period [41].

Clinical and laboratory tests, such as monitoring GH and IGF-I levels, provide critical information about disease activity and treatment efficacy. However, these tests alone may not sufficiently capture all aspects of cardiovascular risk in acromegaly patients. While IGF-I levels can indicate the severity of acromegaly and correlate with some cardiovascular risks, they do not directly assess cardiovascular health. Other factors such as blood pressure, lipid profiles, and blood glucose levels are also essential but may not fully capture the impact of GH excess on the vascular system. Available diagnostic methods often require the involvement of additional cardiovascular specialists, and thus additional financial resources, to complete cardiometabolic evaluation of this group of patients. This further prolongs the diagnostic and therapeutic process in acromegaly. The search for new diagnostic tools to identify cardiovascular complications, including CPP or MR-proADM analysis, serves to improve and facilitate the diagnostic pathway in acromegaly. These markers may reflect endothelial dysfunction, vascular remodeling, and inflammation, which are critical in the pathophysiology of cardiovascular diseases [20,22,27,28]. Therefore, they could provide additional insights into cardiovascular risk, even if they are not yet fully validated for routine clinical use. They also could give this information about cardiovascular risk that standard tests might miss. In addition, in the assessment of these biomarkers added to commonly used tests such as NT-proBNP or troponins, provide more complete prognostic information. The monitoring cardiovascular health in acromegaly by using multiple markers is very advisable. Early identification of cardiovascular complications and the inclusion of appropriate treatment is expected to extend the life expectancy of patients with acromegaly.

Our study evaluated two biomarkers, CPP and MR-proADM, in acromegaly patients and a CG to explore their potential relevance to these disorders. Notably, the association of these markers in acromegaly has not been previously investigated.

CPP, through equimolar secretion of arginine vasopressin (AVP), is a valuable diagnostic and prognostic marker for CVD. Numerous studies have examined its utility in the rapid exclusion of acute myocardial infarction, as well as in predicting mortality rates associated with heart failure and stroke [19,42,43,44]. Additionally, numerous reports in the literature recognize CPP as a marker of diabetic insipidus (DI) [45,46]. In central DI, the most common condition is insufficient AVP secretion from the posterior pituitary gland, which is caused by damage to the of the posterior pituitary gland or the medial hypothalamic protrusion. The most common acquired causes are trauma, pituitary surgery, neoplastic, vascular, autoimmune, infectious, or granulomatous diseases [21,45]. Therefore, CPP may indirectly serve as a biomarker for posterior pituitary lesion. Furthermore, it has been studied as a diagnostic tool for the syndrome of inappropriate antidiuretic hormone secretion (SIADH) and other causes of hyponatremia. However, it is not considered a reliable parameter for these conditions due to its limited sensitivity [47]. Moreover, elevated CPP levels upon admission to the intensive care unit predict short- and long-term mortality, indicating its potential application in risk stratification of patients with severe clinical conditions [48].

Enhörning et al. observed increased CPP levels in patients with new-onset diabetes mellitus [49]. Furthermore, plasma concentrations of this biomarker have shown a positive correlation with the significant (cardiovascular and renal) complications of diabetes mellitus [47]. Increased CPP levels may be caused by chronic inflammation and elevated circulating inflammatory cytokines accompanying insulin resistance, impaired glucose tolerance, hyperinsulinemia, diabetes mellitus, and components of metabolic syndrome (hypertension, abdominal obesity, dyslipidemia, elevated TG, and low HDL cholesterol) [50,51]. CPP has proven to be a stable and easy-to-measure surrogate marker for AVP and has shown great potential in clinical practice.

CPP concentrations were not significantly different between the study group and CG in the current study. Lewandowski et al. reported that patients with a history of pituitary disease had lower CPP secretion after corticotrophin-releasing hormone (CRH) stimulation, even in the absence of clinically significant abnormalities in the adrenocorticotrophin hormone (ACTH)-cortisol axis [52]. Reduced CPP secretion has also been documented in subjects with mild pituitary dysfunction during the insulin tolerance test (ITT) [53] and the glucagon stimulation test (GST) [54]. These findings suggest that CPP may be a sensitive marker of anterior pituitary lesions.

Among our patients with acromegaly, most (up to 90%) underwent neurosurgery, while 15% received complementary radiation therapy. These therapeutic interventions on the pituitary gland may have caused local tissue damage. Consequently, despite the absence of a requirement for hormone replacement therapy in these patients, we did not observe the expected elevation in CPP levels within the study group, though we found a positive correlation between GH and CPP levels in the patients with acromegaly. According to a study by Sjöström et al., patients treated with GH for six months showed a significant increase in IGF-I and CPP after three months, which was maintained over the six-month observation period. However, the correlation of IGF-I and CPP was poor—most likely a result of different individual IGF-I levels [55]. In our study, we obtained a negative correlation between fasting glucose concentrations and CPP levels in acromegaly patients but found no correlation between the other parameters of carbohydrate and lipid metabolism and CPP concentrations, including in the CG. In contrast, Sjöström et al. reported increased hemoglobin A1c (HbA1c) and decreased LDL cholesterol, with a concomitant CPP increase in patients treated with GH [54], whereas an association was revealed between CPP, metabolic syndrome, BMI, plasma glucose levels, insulin resistance, TG, and HDL cholesterol in the American population [56]. Our results are most likely due to the different degrees of metabolic and hormonal alignment in the pituitary somatotropic axis of the patients.

ADM has anti-inflammatory, vasodilator, vascular permeability regulation, and angiogenesis properties, and its levels increase in various pathological conditions, such as sepsis, severe infections (including COVID-19), acute ischemic stroke, and vascular cognitive impairment [57,58,59]. MR-proADM, a more stable form of ADM, has been studied as a prognostic marker in acute and chronic heart failure, after acute myocardial infarction, and in chronic kidney disease [24,27,60]. A member of the adipokine family, the concentration of this biomarker was significantly higher in obese adolescents than in normal-weight contemporaries. In addition, MR-proADM levels have been associated with BMI, fat mass, insulin levels, HOMA-IR, total cholesterol, and LDL cholesterol, suggesting its significant impact on obesity development [61].

Our analysis showed no significant differences between the study and control groups in terms of MR-proADM concentrations and found no correlation between this biomarker and pituitary somatotropic axis hormone levels in patients with acromegaly. However, we found a positive correlation between LDL and HDL concentrations and MR-proADM levels in patients with acromegaly. The relationship between these lipoproteins and MR-proADM is complex and reflects a multifaceted interaction between lipid metabolism and vascular health. HDL cholesterol, through its involvement in reverse cholesterol transport and anti-inflammatory effects, is thought to protect against cardiovascular disease [62]. The positive correlation of this lipoprotein with MR-proADM may indicate that elevated HDL cholesterol may be part of a compensatory response to vascular stress or inflammation as the body attempts to offset adverse cardiovascular effects. Referred to as the atherogenic, oxidized form of LDL cholesterol, it contributes to the formation of atherosclerotic plaques in the arteries [63]. Elevated LDL cholesterol levels may positively correlate with MR-proADM in systemic inflammation or insulin resistance, suggesting that higher lipid levels may occur in a state of endothelial dysfunction. Therefore, the positive correlation between HDL cholesterol, LDL cholesterol, and MR-proADM may be due to underlying mechanisms such as inflammation, endothelial dysfunction, and adaptive responses to vascular stress [30,64]. However, the KORA F4 study indicated an atherosclerotic effect of MR-proADM. Receptors for ADM were found to be present in adipose tissue, and the action of this adipokine is to reduce lipolysis by inhibiting beta-adrenergic pathways in adipose tissue, which results in changes in plasma lipid concentrations (increased MR-proADM levels have been associated with increased triglyceride concentrations and decreased HDL cholesterol concentrations) [65]. This illustrates how complex and interrelated lipid profiles and markers of cardiovascular disease can be.

In our study, we detected no correlation between carbohydrate metabolism parameters and MR-proADM levels in the study or control groups. In contrast, Sujana et al. showed that elevated MR-proADM concentrations were associated with an increased risk of insulin resistance (higher fasting insulin levels and HOMA-IR) compared to participants without diabetes [66]. The results of our study suggest that this biomarker is not an optimal indicator of metabolic disorders in patients with acromegaly. Due to the small sample size, which is the main limitation of our study, the above results are preliminary and require further research. Moreover, MR-proADM expression is observed in numerous anatomical sites, and interpreting the levels of these biomarkers may be influenced by various factors. Furthermore, there is a lack of studies comparing the association between such biochemical parameters and MR-proADM in patients with acromegaly.

The most common cardiovascular complication in acromegaly patients was hypertension, occurring in more than one in two subjects (52.83%). In a study by Harsha et al., the disease affected almost one in three patients with acromegaly. The prevalence of hypertension in this group of patients varies, ranging from 18 to 60%, with a mean prevalence of around 35% [4,39]. In the large ACROSTUDY involving 2090 acromegaly patients treated with pegvisomant, hypertension occurred at a rate of 56% at the start of the study and increased to 64% during the observation period. At the time of study inclusion, patients who belonged to the hypertensive group were older at initial acromegaly diagnosis, had a higher BMI, and more frequent cardiovascular risk factors (smoking, other CVD, diabetes, and hyperlipidemia). In addition, they had more comorbidities with pituitary deficiency [67]. Among our patients, mild valvular regurgitation, myocardial hypertrophy, and atrial cavity enlargement were the most common abnormalities in echocardiograms. According to Colao, the most common characteristic of acromegalic cardiomyopathy is concentric biventricular cardiac hypertrophy, the determinants of which are aging and long active disease duration (GH/IGF-I excess) [68].

Among the carbohydrate and lipid metabolism disorders in our study group, hyperlipidemia predominated with a prevalence of 68%, while other metabolic complications occurred at a similar frequency of around 30%. A Turkish study of acromegaly patients found that hypertriglyceridemia and low HDL cholesterol were the most common lipid metabolic disorders in this group of patients, occurring with a frequency of 42% and 49%, respectively. However, diabetes mellitus and prediabetes affected a similar proportion of acromegaly patients as our study [69]. Moreover, Matsubayashi et al. observed that the most common comorbidities among acromegaly patients were hypertension (43%), diabetes (37%), and hyperlipidemia (27%) [70], which occurred at a lower incidence compared to our study group.

In active acromegaly, excess GH and IGF-I cause changes in body composition and abnormalities in glucose and lipid metabolism. In these patients, as a result of accelerated lipolysis, the amount of free fatty acids in circulation increases, resulting in the development of hepatic and extrahepatic insulin resistance, adipose tissue inflammation, and changes in fat distribution, known as acromegalic lipodystrophy [71]. This body composition pattern, caused by adipose tissue dysregulation, is characterized by a decrease in total body fat, especially visceral fat deposition, a decrease in intrahepatic lipid, and an increase in muscle lipid deposition. It should be added that biochemical remission after treatment of acromegaly reverses the pattern of lipodystrophy. Moreover, pegvisomant therapy exacerbates hepatic steatosis [72].

Among our patients, we observed that atherogenic dyslipidemia was significantly more frequent in AA than the CG, though we did not notice a significant effect of acromegaly activity for other metabolic complications. Moreover, we found atherogenic dyslipidemia to be more common in patients with acromegaly due to pituitary macroadenoma compared to the controls. Can et al. reported that, among acromegaly complications, diabetes was more common in those with active disease than in the group of well-controlled patients [69]. In addition to diabetes, Carmichael et al. observed a significantly higher prevalence of hypertension in patients with AA [73]. Reid et al. showed that AA is associated with lower insulin sensitivity, reduced body fat, and lower CRP levels than controlled acromegaly, and %ULN IGF-I more strongly predicts insulin sensitivity than GH. Moreover, they observed no association between blood pressure, lipidograms, and disease activity [74].

According to Powlson et al., neurosurgical treatment of acromegaly reduced left ventricular mass, improved diastolic function, and decreased diastolic blood pressure [10]. A study by Yen et al. showed significant reductions in HbA1c and TG after transsphenoidal adenomectomy, regardless of the hormonal status of acromegaly patients. However, other cardiovascular risk factors (systolic blood pressure, total cholesterol, HDL cholesterol, fasting glucose, and TG) improved after neurosurgical treatment in patients with high cardiovascular risk before surgery [75]. An analysis by Reyes-Vidal et al. showed improvements in cardiometabolic markers such as insulin resistance, HDL cholesterol levels, and systolic blood pressure after achieving acromegaly remission following surgical treatment. On the other hand, an increase in patient body weight, a higher prevalence of central obesity, and an increase in total ghrelin levels were observed after this treatment [76], demonstrating the importance of optimizing therapy after achieving remission of the underlying disease.

Sharma et al. and Heidarpour et al. indicated that therapy with first-generation somatostatin analogs (SSA—octreotide and lanreotide) through the control of excess GH and IGF-I and direct actions of SSA (somatostatin receptor types 1, 2, 4 and 5) on the heart and vessels, improved cardiovascular parameters (blood pressure, heart rate, systolic and diastolic function, exercise tolerance, left ventricular mass, QT interval duration, and arrhythmia rate) in patients who have not achieved complete biochemical control of the disease [11,77]. In addition, effective control of AA with this medication improves glucose and lipid metabolism, although impaired insulin secretion leading to further increases in glucose levels may occur at the beginning of this pharmacotherapy. However, this effect subsides over the duration of the treatment (especially in elderly patients). Nonetheless, diabetes mellitus may develop and force the discontinuation of treatment, though this is rare [78]. Moreover, long-term therapy with pegvisomant, a GH receptor antagonist, normalizes IGF-I, improves acromegalic cardiomyopathy by reducing myocardial hypertrophy, and enhances diastolic and systolic function, resulting in the partial or complete reversal of heart failure [79].

Our analysis showed significantly higher fasting glucose and insulin levels in patients with acromegaly compared to controls and an inverse association for the HOMA-IR index. The two groups did not differ in other carbohydrate and lipid metabolism parameters. The reason for the results may be the presence of metabolic diseases and their suboptimal compensation among control subjects. In contrast, Dimopoulou et al. observed significantly lower fasting glucose concentrations among acromegaly patients than the CG. Moreover, the study group had lower levels of total cholesterol and TG and higher levels of HDL cholesterol. Furthermore, the authors found that patients with biochemically controlled acromegaly had unfavorable cardiovascular parameters compared to controls, which could be due to overtreatment causing relative GH deficiency and hypopituitarism of other axes, insufficient exercise resulting from joint pain, and side effects of the drugs used (i.e., pegvisomant increases body weight and body fat measurements) [80].

Lin et al. compared three groups of patients with either GH deficiency after definitive treatment of acromegaly, sufficient levels of GH after acromegaly treatment, or AA, and observed a positive correlation between IGF-I levels and fasting blood glucose, blood glucose on 120 min OGTT, HOMA-IR, and HbA1c. In contrast, there were no significant associations between IGF-I levels and total cholesterol, LDL cholesterol, or TG. In addition, they showed that GH deficiency after definitive treatment of acromegaly negatively influenced body composition and inflammatory biomarkers of cardiovascular risk but did not affect carbohydrates, lipids, or other markers of cardiovascular risk [81]. AA is associated with increased mortality from comorbidities. Recent decades have brought significant advances in treating this disease and its complications, reducing mortality rates to levels comparable to the general population [82].

Both biomarkers we studied (CPP and MR-proADM) are involved in inflammatory stress, which can cause CVD [29,31]. In acromegaly, cardiovascular complications are a major cause of mortality [4]. Inflammation is both a cause and an exacerbator of CVD. Diagnostic and prognostic biomarkers such as sirtuins, microRNA, ST2 protein, apolipoprotein E, and adiponectin, which have been studied in recent decades, are expressed locally in inflammation target tissue and then released into the peripheral blood. They can be used to predict adverse cardiovascular events and worse prognosis in patients [83]. Therefore, it is important to search for new biomarkers valuable in the diagnostic and therapeutic process.

It is also worth emphasizing that acromegaly causes systemic complications, including from the gastrointestinal tract. By slowing the orocecal transit time (OCTT) and reducing the normal removal of bacteria from the small intestine, resulting from elongation, intestinal dilation, and autonomic dysfunction, patients with acromegaly often suffer from small intestinal bacterial overgrowth (SIBO) [84]. Resmini et al. showed that patients with acromegaly had an increased incidence of SIBO (43.9% versus 3.3%) and had significantly delayed OCTT (169.53 ± 8.15 versus 107.25 ± 6.56 min) compared to controls. The consequences of untreated SIBO, in addition to gastrointestinal complaints (such as bloating, abdominal pain, chronic diarrhea, a feeling of fullness in the abdomen, and constipation), include weight loss, malnutrition, edema, vitamin deficiency symptoms, and skin lesions [85]. Therefore, patients with acromegaly require a holistic diagnostic approach.

In the context of the diagnosis and complications of acromegaly, it is also important to mention the genetic background of this endocrinopathy, which causes clinical implications. In addition, genetic testing identifies family members at an earlier stage of the disease, leading to improved long-term treatment outcomes. Based on the genetic background, acromegaly is divided into isolated and syndromic forms. Isolated acromegaly refers to molecular defects that exclusively predispose individuals to PitNET, including familial isolated pituitary adenomas (FIPA) and sporadic defects without an inherited predisposition [86]. In most cases, somatotropinomas result from somatic mutations in the *GNAS* gene. Patients with this mutation are diagnosed at a relatively older age and have smaller and less invasive tumors at the time of diagnosis. Most studies suggest that *GNAS* mutations are associated with dense somatotrophic tumors, and the efficacy of first-generation SSA is higher [87]. In contrast, syndromic forms (about 5% of all pituitary tumors) include acromegaly as part of a syndromic disease accompanied by other symptoms, often tumors of other endocrine organs, such as multiple endocrine neoplasia type 1 (MEN1), multiple endocrine neoplasia type 4 (MEN4), Carney complex, McCune–Albright syndrome, phaeochromocytoma/paraganglioma (PPGL)-pituitary adenoma association (3PA), Neurofibromatosis type 1, X-linked acrogigantism (X-LAG), or Tuberous Sclerosis Complex (TSC) [88]. In these cases, tumors generally have a familial occurrence, are observed in young patients, and are characterized by accelerated growth, invasion, and resistance to surgery and medical therapy, especially first-generation somatostatin analogs, making them more clinically challenging [87,89].

## 5. Conclusions

In conclusion, this study is the first to analyze CPP and MR-proADM biomarkers in patients with acromegaly. Our findings indicate that, although these biomarkers are valuable in diagnosing other conditions, they do not serve as reliable indicators for diagnosing or monitoring acromegaly complications. Given the small and heterogeneous patient sample—a notable limitation—these findings are preliminary, emphasizing the need for further studies with larger cohorts. Additionally, the gradual onset and often delayed diagnosis of acromegaly prevent an accurate assessment of long-term GH exposure, which could influence results. A prospective analysis would likely provide additional insights. Acromegaly is associated with the prevalence of many complications, especially cardiovascular and metabolic, which requires the diagnostic vigilance necessary for a proper therapeutic process. Identifying non-invasive prognostic markers to detect increased cardiometabolic risk in acromegaly patients could support more targeted therapy, slow the progression of the disease and its complications, and improve patients’ quality of life.

## Figures and Tables

**Table 1:** Characteristics of the study (acromegaly) and control groups.

Parameters ($\overset{®}{x}$ ± SD/Me (Q1-Q3)/ %)	Acromegaly Group	Control Group	Test Name	df	Test Value	*p*
Sex (F %/M %)	56.60/43.40	69.23/30.77	χZ^2^	1.00	1.17	0.280
Age (years)	56.00 (43.00–67.00)	60.00 (50.00–70.00)	*W*	63.67	2.84	0.006
Height (cm)	170.35 ± 9.80	165.00 ± 6.22	Z	-	−1.25	0.211
Weight (kg)	86.50 (75.00–100.50)	68.00 (60.00–82.50)	Z	-	3.12	0.002
BMI (kg/m^2^)	29.75 (26.50–32.92)	24.80 (23.34–31.80)	Z	-	2.30	0.021
Systolic pressure (mm/Hg)	129.70 (120.0–135.00)	124.80 (120.00–140.00)	Z	-	−0.63	0.530
Diastolic pressure (mm/Hg)	81.04 (70.00–85.00)	80.00 (80.00–86.00)	Z	-	0.47	0.568
CPP (pg/mL)	65.11 (52.09–87.01)	60.80 (44.76–78.41)	Z	-	1.28	0.201
MR-proADM (ng/L)	96.67 (68.00–149.13)	86.01 (72.38–183.06)	Z	-	−0.01	0.996
GH (ng/mL)	0.97 (0.45–2.53)	0.27 (0.15–2.55)	Z	-	−1.99	0.047
IGF-I × ULN	0.69 (0.59–1.06)	0.49 (0.37–0.66)	Z	-	−2.61	0.009
Glucose (mg/dL)	95.00 (87.00–106.00)	89.00 (84.00–96.00)	Z	-	2.08	0.037
Glucose at 120 min OGTT (mg/dL)	96.00 (72.00–119.00)	89.00 (84.00–96.00)	t	49.00	0.95	0.348
Insulin (µIU/mL)	6.52 (2.86–11.80)	3.22 (6.37–14.70)	Z	-	−2.02	0.043
HOMA-IR	0.36 (0.33–0.41)	2.10 (1.56–3.13)	Z	-	−1.92	0.054
QUICKI	0.36 (0.33–0.41)	0.34 (0.32–0.36)	Z	-	1.82	0.069
Total cholesterol (mg/dL)	189.94 ± 41.82	189.08 ± 42.87	t	75.00	0.83	0.934
LDL cholesterol (mg/dL)	115.47 ± 36.24	108.00 ± 38.70	t	77.00	0.15	0.879
HDL cholesterol (mg/dL)	48.00 (46.00–60.00)	54.50 (41.50–60.50)	Z	-	−0.62	0.540
TG (mg/dL)	119.00 (85.00–129.00)	122.00 (84.00–146.00)	Z	-	−1.00	0.320
Castelli 1	3.61 (3.08–4.21)	3.42 (2.75–4.48)	Z	-	0.81	0.420
Castelli 2	2.12 (1.78–2.64)	1.89 (1.51–2.78)	Z	-	0.89	0.380
API	0.37 (0.16–0.46)	0.35 (0.16–0.53)	Z	-	0.05	0.950
AC	2.16 (2.08–3.21)	2.42 (1.75–3.48)	Z	-	0.81	0.420

$\overset{®}{x}$ ± SD—mean and standard deviation; Me (Q1–Q3)—median and quartiles; χZ^2^—test statistic of the correlation test; W—Welch’s *t*-test value; Z—Mann–Whitney-U test value (independent Wilcoxon rank sum test); t—Student’s *t*-test value; df—degrees of freedom; *p*—level of statistical significance. Assumed α = 0.05. F—female; M—male; BMI—body mass index; CPP—copeptin; MR-proADM—mid-regional proadrenomedullin; GH—growth hormone; IGF-I—insulin-like growth factor 1; ULN—upper limit of normal; OGTT—oral glucose tolerance test; HOMA-IR—homeostatic model assessment for insulin resistance; QUICKI—quantitative insulin sensitivity check index; LDL cholesterol—low-density lipoprotein cholesterol; HDL cholesterol—high-density lipoprotein cholesterol; TG—triglycerides; API—atherogenic index of plasma; AC—atherogenic coefficient.

**Table 2:** Correlation of hormones with biomarkers in the study group (acromegaly).

Variable	N	t(N-2)	ρ	*p*
Hormones vs. CPP				
IGF-I × ULN vs. CPP (pg/mL)	53	−0.44	−0.06	0.662
GH (ng/mL) vs. CPP (pg/mL)	51	1.93	0.27	0.060
Hormones vs. MR-proADM				
IGF-I × ULN vs. MR-proADM (ng/L)	53	−1.13	−0.16	0.265
GH (ng/mL) vs. MR-proADM (ng/L)	51	−0.42	−0.06	0.675

N—number of observations; t(N-2)—Student’s *t*-test statistic; ρ—Spearman’s correlation coefficient indicating the monotonic component of the relationship; *p*—level of statistical significance. Assumed α = 0.05.

**Table 3:** Correlations of carbohydrate and lipid metabolism parameters with CPP in the study group (acromegaly) and the control group.

Variable	N	tau-c/ρ	Z/t(N-2)	*p*
Acromegaly group				
Carbohydrate metabolism parameters vs. CPP				
Glucose (mg/dL) vs. CPP (pg/mL)	49	tau-c = −0.11	Z = −0.14	0.025
Glucose at 120 min OGTT (mg/dL) vs. CPP (pg/mL)	37	R = −0.05	t(N-2) = −0.17	0.869
Insulin (μlU/mL) vs. CPP (pg/mL)	45	R = 0.04	t(N-2) = 0.20	0.846
HOMA-IR vs. CPP (pg/mL)	43	R = −0.09	t(N-2) = −0.41	0.687
QUICKI vs. CPP (pg/mL)	42	R = 0.10	t(N-2) = 0.41	0.687
Lipid metabolism parameters vs. CPP				
Total cholesterol (mg/dL) vs. CPP (pg/mL)	53	R = 0.27	t(N-2) = 1.33	0.196
LDL cholesterol (mg/dL) vs. CPP (pg/mL)	53	tau-c = −0.10	Z = −1.05	0.295
HDL cholesterol (mg/dL) vs. CPP (pg/mL)	53	tau-c = −0.10	Z = −1.03	0.301
TG (mg/dL) vs. CPP (pg/mL)	53	tau-c = 0.09	Z = 0.90	0.368
Castelli 1 vs. CPP (pg/mL)	53	R = 0.09	t(N-2) = 0.44	0.665
Castelli 2 vs. CPP (pg/mL)	53	R = 0.06	t(N-2) = 0.27	0.790
API vs. CPP (pg/mL)	53	R = 0.01	t(N-2) = 0.04	0.968
AC vs. CPP (pg/mL)	53	R = 0.09	t(N-2) = 0.44	0.665
Control group				
Carbohydrate metabolism parameters vs. CPP				
Glucose (mg/dL) vs. CPP (pg/mL)	23	tau-c = 0.12	Z = 0.83	0.409
Glucose at 120 min OGTT (mg/dL) vs. CPP (pg/mL)	14	tau-c = −0.01	Z = −0.06	0.956
Insulin (mg/dL) vs. CPP (pg/mL)	22	R = 0.04	t(N-2) = 0.20	0.846
HOMA-IR vs. CPP (pg/mL)	21	R = −0.10	t(N-2) = −0.41	0.687
QUICKI vs. CPP (pg/mL)	21	R = 0.09	t(N-2) = 0.41	0.687
Lipid metabolism parameters vs. CPP				
Total cholesterol (mg/dL) vs. CPP (pg/mL)	24	R = 0.27	t(N-2) = 1.33	0.196
LDL cholesterol (mg/dL) vs. CPP (pg/mL)	26	R = 0.25	t(N-2) = 1.26	0.221
HDL cholesterol (mg/dL) vs. CPP (pg/mL)	24	tau-c = 0.11	Z = 0.73	0.468
TG (mg/dL) vs. CPP (pg/mL)	25	R = 0.07	t(N-2) = 0.33	0.743
Castelli 1 vs. CPP (pg/mL)	24	R = 0.09	t(N-2) = 0.44	0.665
Castelli 2 vs. CPP (pg/mL)	24	R = 0.06	t(N-2) = 0.27	0.790
API vs. CPP (pg/mL)	24	R = 0.10	t(N-2) = 0.47	0.642
AC vs. CPP (pg/mL)	24	R = 0.09	t(N-2) = 0.44	0.665

N—number of observations; ρ—Spearman’s rank correlation coefficient indicating the monotonic component of the relationship; tau-c—Stewart–Kendall’s tau-c rank correlation coefficient; *p*—level of statistical significance. Assumed α = 0.05.

**Table 4:** Correlations of carbohydrate and lipid metabolism parameters with MR-proADM in the study group (acromegaly) and the control group.

Variable	N	tau-c/ρ	Z/t(N-2)	*p*
Acromegaly group				
Carbohydrate metabolism parameters vs. MR-proADM				
Glucose (mg/dL) vs. MR-proADM (ng/L)	49	tau-c = −0.01	Z = −0.05	0.958
Glucose at 120 min OGTT (mg/dL) vs. MR-proADM (ng/L)	37	R = 0.35	t(N-2) = 1.30	0.220
Insulin (μlU/mL) vs. MR-proADM (ng/L)	45	R = −0.16	t(N-2) = −0.72	0.482
HOMA-IR vs. MR-proADM (ng/L)	43	R = −0.10	t(N-2) = −0.43	0.674
QUICKI vs. MR-proADM (ng/L)	42	R = 0.10	t(N-2) = 0.43	0.674
Lipid metabolism parameters vs. MR-proADM				
Total cholesterol (mg/dL) vs. MR-proADM (ng/L)	53	R = −0.01	t(N-2) = 0.01	0.998
LDL cholesterol (mg/dL) vs. MR-proADM (ng/L)	53	tau-c = 0.24	Z = 2.56	0.010
HDL cholesterol (mg/dL) vs. MR-proADM (ng/L)	53	tau-c = 0.23	Z = 2.40	0.017
TG (mg/dL) vs. MR-proADM (ng/L)	53	tau-c = −0.05	Z = −0.55	0.579
Castelli 1 vs. MR-proADM (ng/L)	53	R = −0.01	t(N-2) = −0.03	0.974
Castelli 2 vs. MR-proADM (ng/L)	53	R = −0.03	t(N-2) = −0.14	0.891
API vs. MR-proADM (ng/L)	53	R = 0.10	t(N-2) = 0.47	0.642
AC vs. MR-proADM (ng/L)	53	R = −0.01	t(N-2) = −0.03	0.974
Control group				
Carbohydrate metabolism parameters vs. MR-proADM				
Glucose (mg/dL) vs. MR-proADM (ng/L)	23	tau-c = −0.04	Z = −0.24	0.811
Glucose at 120 min OGTT (mg/dL) vs. MR-proADM (ng/L)	14	tau-c = 0.28	Z = 1.38	0.166
Insulin (mg/dL) vs. MR-proADM (ng/L)	22	R = −0.16	t(N-2) = −0.72	0.482
HOMA-IR vs. MR-proADM (ng/L)	21	R = −0.08	t(N-2) = −0.41	0.687
QUICKI vs. MR-proADM (ng/L)	21	R = 0.10	t(N-2) = 0.43	0.674
Lipid metabolism parameters vs. MR-proADM				
Total cholesterol (mg/dL) vs. MR-proADM (ng/L)	24	R = 0.01	t(N-2) = 0.01	0.998
LDL cholesterol (mg/dL) vs. MR-proADM (ng/L)	26	R = −0.08	t(N-2) = −0.41	0.684
HDL cholesterol (mg/dL) vs. MR-proADM (ng/L)	24	tau-c = −0.09	Z = −0.63	0.531
TG (mg/dL) vs. MR-proADM (ng/L)	25	R = 0.07	t(N-2) = 0.33	0.743
Castelli 1 vs. MR-proADM (ng/L)	24	R = −0.01	t(N-2) = −0.03	0.974
Castelli 2 vs. MR-proADM (ng/L)	24	R = −0.03	t(N-2) = −0.14	0.891
API vs. MR-proADM (ng/L)	24	R = 0.10	t(N-2) = 0.47	0.642
AC vs. MR-proADM (ng/L)	24	R = −0.01	t(N-2) = −0.03	0.974

N—number of observations; ρ—Spearman’s rank correlation coefficient indicating the monotonic component of the relationship; tau-c—Stewart–Kendall’s tau-c rank correlation coefficient; *p*—level of statistical significance. Assumed α = 0.05.

**Table 5:** Comorbidities in acromegaly patients and controls.

	A (n = 53) (N/%)	CG (n = 26) (N/%)	Test Name	df	Test Value	*p*
Cardiovascular complications						
Hypertension	28 (52.83%)	15 (65.22%)	χZ^2^	1.00	1.00	0.317
Arrhythmia	8 (15.09%)	4 (20.00%)	Fe	-	-	0.725
Changes in echocardiograms	15 (28.30%)	3 (15.00%)	Fe	-	-	0.363
Heart failure	2 (3.77%)	0 (0.00%)	Fe	-	-	1.000
Coronary artery disease	1 (1.89%)	3 (15.00%)	Fe	-	-	0.060
Arterial and capillary disease	10 (18.87%)	3 (15.00%)	Fe	-	-	1.000
History of embolism	1 (1.89%)	0 (0.00%)	Fe	-	-	1.000
History of stroke	3 (5.66%)	0 (0.00%)	Fe	-	-	0.557
Metabolic complications						
Hyperlipidemia	36 (67.92%)	18 (81.82%)	χZ^2^	1.00	1.49	0.222
Prediabetes	14 (26.42%)	4 (20.00%)	Fe	-	-	0.763
Diabetes	15 (28.30%)	5 (23.81%)	χZ^2^	1.00	0.15	0.695
Insulin resistance	15 (34.88%)	10 (47.62%)	χZ^2^	1.00	0.96	0.327
Atherogenic dyslipidemia	15 (28.30%)	8 (33.33%)	χZ^2^	1.00	0.20	0.655

A—acromegaly group (study group); CG—control group; N—number of observations; test name: χZ^2^—the value of the correlation test statistic; Fe—the value of the test statistic for Fisher’s exact test; df—degrees of freedom; *p*—level of statistical significance. Assumed α = 0.05.

**Table 6:** Effects of acromegaly activity and pituitary tumor size on lipid metabolism disorders in the acromegaly group.

Comorbidity				Test Name	*p*
Lipid Metabolism Disorders					
Acromegaly activity and lipid metabolism disorders					
Acromegaly activity and atherogenic dyslipidemia				F	0.046
Acromegaly activity	Atherogenic dyslipidemia	Absence of atherogenic dyslipidemia	Total		
AA	6 (40.00%)	5 (13.16%)	11		
CoA	4 (26.67%)	23 (60.53%)	27		
CuA	5 (33.33%)	10 (26.32%)	15		
	15	38	53		
Pituitary tumor size and lipid metabolism disorders					
Pituitary tumor size and atherogenic dyslipidemia				Fe	0.046
Pituitary tumor size	Atherogenic dyslipidemia	Absence of atherogenic dyslipidemia	Total		
Microadenoma	0 (0.00%)	10 (26.32%)	10		
Macroadenoma	15 (100.00%)	28 (73.68%)	43		
	15	38	53		

AA—active acromegaly; CoA—controlled acromegaly; CuA—cured acromegaly; test name: F—Fisher–Freeman–Halton test statistic; Fe—Fisher’s exact test statistic; *p*—level of statistical significance. Assumed α = 0.05.

## Data Availability

The original contributions presented in the study are included in the article; further inquiries can be directed to the corresponding author.
